# Correction to: SPIKE-1: A Randomised Phase II/III trial in a community setting, assessing use of camostat in reducing the clinical progression of COVID-19 by blocking SARS-CoV-2 Spike protein-initiated membrane fusion

**DOI:** 10.1186/s13063-022-06266-0

**Published:** 2022-04-21

**Authors:** Sarah Halford, Susan Wan, Ilaria Dragoni, Julie Silvester, Bobojon Nazarov, Daniel Anthony, Suzie Anthony, Emma Ladds, John Norrie, Kevin Dhaliwal, and the CDD SPIKE-1 Project Team

**Affiliations:** 1 Cancer Research UK Centre for Drug Development, London, UK; 2 Latus Therapeutics, London, UK; 3 University of Oxford, Oxford, UK; 4 Oxford University Hospitals NHS Foundation Trust, Oxford, UK; 5 Nuffield Department of Primary Care Health Sciences, University of Oxford, Oxford, UK; 6 The University of Edinburgh, Edinburgh, UK

**Correction to: Trials 22, 550 (2021)**

**https://doi.org/10.1186/s13063-021-05461-9**

Following the publication of the original article [1], we were notified that the Flu-iiQ™ table included in the supplementary material of original publication needs to be deleted. The authors were not at liberty to publish the table in full as a number of the items listed are confidential and commercially sensitive. For full details of the Flu-iiQ™ questionnaire please contact Measured Solutions P/L, PO Box 5127, Alphington VIC 3079, Australia (measuredsolutions.com.au).

The original article has been corrected.

## References

[1] Halford (2021). SPIKE-1: A Randomised Phase II/III trial in a community setting, assessing use of camostat in reducing the clinical progression of COVID-19 by blocking SARS-CoV-2 Spike protein-initiated membrane fusion. Trials..

